# Characterization of Volatile Component Changes in Jujube Fruits during Cold Storage by Using Headspace-Gas Chromatography-Ion Mobility Spectrometry

**DOI:** 10.3390/molecules24213904

**Published:** 2019-10-30

**Authors:** Lvzhu Yang, Jie Liu, Xinyu Wang, Rongrong Wang, Fang Ren, Qun Zhang, Yang Shan, Shenghua Ding

**Affiliations:** 1 Longping Branch Graduate School, Hunan University, Changsha 410125, China; ylzhu0115@163.com (L.Y.); jiel95712@163.com (J.L.); wxy25994@163.com (X.W.); 2 Provincial Key Laboratory for Fruits and Vegetables Storage Processing and Quality Safety, Agricultural Product Processing Institute, Hunan Academy of Agricultural Sciences, Changsha 410125, China; zqun208@163.com (Q.Z.); sy6302@sohu.com (Y.S.); 3 College of Food Science and Technology, Hunan Agricultural University, Changsha 410128, China; 4 G.A.S. Department of Shandong Hanon Science Instrument Co., Ltd., Jinan 253000, China; amanda@hanon.cc

**Keywords:** jujube fruits, volatile components, headspace-gas chromatography-ion mobility spectrometry (HS-GC-IMS), cold storage, principal component analysis (PCA)

## Abstract

Volatile components in jujube fruits from *Zizyphus jujuba* Mill. *cv*. Dongzao (DZ) and *Zizyphus jujuba* Mill. *cv*. Jinsixiaozao (JS) were analyzed under different cold storage periods via headspace-gas chromatography-ion mobility spectrometry (HS-GC-IMS). Results identified 53 peaks that corresponded to 47 compounds and were mostly alcohols, aldehydes, esters, and ketones. Differences in the volatile components of jujube fruits were revealed in topographic plots and fingerprints. For DZ, 3-pentanone was the characteristic component of fresh fruits. After storage for 15 days, dipropyl disulfide became the most special substance. Moreover, when stored for 30 and 45 days, the fruits had some same volatile components, like 2-pentyl furan and diallyl sulfide. However, for DZ stored for 60 days, esters were the prominent constituent of the volatile components, simultaneously, some new alcohols appeared. For JS, 2-ethyl furan was the representative of fresh fruits, and 2-butoxyethanol content was the most abundant after 15 and 30 days of storage. Different from that in DZ, the content of ester in JS increased after storage for 45 days. Substances such as amyl acetate dimer, methyl salicylate, and linalool greatly contributed to the jujube flavor during the late storage period. Principal component analysis (PCA) showed that fresh samples and refrigerated fruits were effectively distinguished. Heat map clustering analysis displayed the similarity of volatile components in different samples and was in accordance with PCA results. Hence, the volatile components of jujube fruits can be readily identified via HS-GC-IMS, and jujube fruits can be classified at different periods based on the difference of volatile components.

## 1. Introduction

Jujube (*Zizyphus jujuba* Mill) tree belongs to the Rhamnaceae family, which is indigenous to China and is distributed worldwide, in places such as Asia, northern Africa, southern Europe, the Middle East, and the southwestern USA. This tree has a history of more than 4000 years, with over 700 cultivars found in China [1,2,3]. Phytochemical analytical studies showed that jujube fruits are rich in nutrients, including fiber, sugars, organic acids, amino acids, vitamins, and trace minerals [4]. They also contain high levels of functional components, such as polysaccharides, triterpene acids, phenolics, and cyclic nucleotides, which exhibit multiple health-promoting properties, such as antioxidant and anti-inflammatory properties and liver protection [5,6,7,8,9]. Recognized for its delicious taste and health beneficial properties, jujube fruits have been consumed for thousands of years as ordinary fruits and Chinese traditional medicine.

In addition to their nutritional and biological activity, jujube fruits are favored as food by consumers due to their unique flavor. Fresh jujube fruits show an extraordinary flavor, but they are highly perishable when not handled properly due to their high moisture content, which leads to the loss of their commercial value. Cold storage is a common means of delaying the deterioration of quality and prolonging shelf-life effectively, which can affect the physicochemical properties of jujube fruits. Liu et al. found that the contents of chlorophyll, ascorbic acid, and soluble solids in fresh-cut jujubes without any treatment were reduced after storage at 4 °C for a certain period [10]. Kou et al. [11] reported that when jujube fruits were stored at 0 °C, the contents of total soluble solids, ascorbic acid, and chlorophylls continuously declined, and the anthocyanin content firstly increased and then decreased. Furthermore, Günther et al. [12] studied kiwifruit and found that cold storage also affected flavor. Simultaneously, changes in post-harvest metabolic and anaerobic environment greatly influence the fruit flavor, anaerobic conditions can enhance the flavor quality of fruits by producing certain aromatic compounds during storage [13]. Aroma profiles are important characteristics to evaluate the quality of fruits. Several studies have reported that the volatile compounds of jujube fruits are affected by many factors, such as growth period [14], extraction methods [15], the load [16], and processing methods [17,18,19,20]. However, few reports have been associated with changes in volatile compounds of jujube fruits during cold storage, which should be considered. The difference in volatile compounds among different periods of jujube fruit cold storage is unclear. Therefore, the purpose of this study was to monitor the changes in flavor and identify its characteristics at different times.

Ion mobility spectrometry (IMS) is an instrumental analytical technique of separating the ions of detected substances based on their ion mobility velocity under atmospheric pressure [21]. It is a convenient and efficient instrument with the advantages of simple sample preparation, easy operation, high sensitivity, and quick analytical speed. Even trace volatile compounds can also be detected in a short time [22,23]. Furthermore, ion mobility notably allows the separation of isomers and isobaric compounds, which cannot be separated even with ultra-high resolution instruments [24]. At first, compounds extracted from the sample enter the ionization chamber directed by a carrier gas, and the analyte is charged after being ionized. Then, a series of reactions occur and reactant ions [H^+^(H_2_O)_n_] are generated. In an IMS instrument, if the proton affinity of the analyte is higher than the proton affinity of water, it will react with the reactant ions. Based on the content of the analyte, their chemical nature or the drift tube temperature, product ions such as protonated monomers or proton-bound dimers were produced [25]. Subsequently, analyte ions enter the drift region through the Bradbury–Nielsen–Shutter. Analyte ions react against the reverse drift gas under the action of the electric field and migrate to the right end to reach the right detector, the drift velocity of ions depends on their charge, mass, and shape [26]. Finally, the Faraday–Plate detects ions and outputs electrical signals. The results are expressed in terms of voltage units. IMS has been used for chemical warfare agents [27], illicit drug detection [28], analysis of explosives [29], and environmental monitoring [30]. The instrument is highly sensitive to high electronegativity and high proton affinity compounds, which can detect a large number of compounds from different chemical families, such as alcohols, aldehydes, aromatics, esters, and ketones [30]. Combining IMS with other instruments is a good way to increase its advantages and produce a good analysis result. In recent years, HS-GC-IMS has been extensively applied to investigate volatile compounds in food science, such as *Tricholoma matsutake* Singer [22], jujube fruits [31], eggs [26], Iberian ham [32,33], and honey [25]. As a consequence, HS-GC-IMS could be used to identify the volatile components of jujube fruits at different periods.

In this study, HS-GC-IMS was used to analyze the variations in the volatile compositions of jujube fruits at different storage periods, and the fingerprints were established to confirm the characteristic substance of each period. The results will provide a new method for studying the flavor of jujube fruits, which will help to rapidly select the best storage time of jujube fruits.

## 2. Results and Discussion

### 2.1. Volatile Components Identification of All Samples at Different Storage Periods

**Figure 1:**
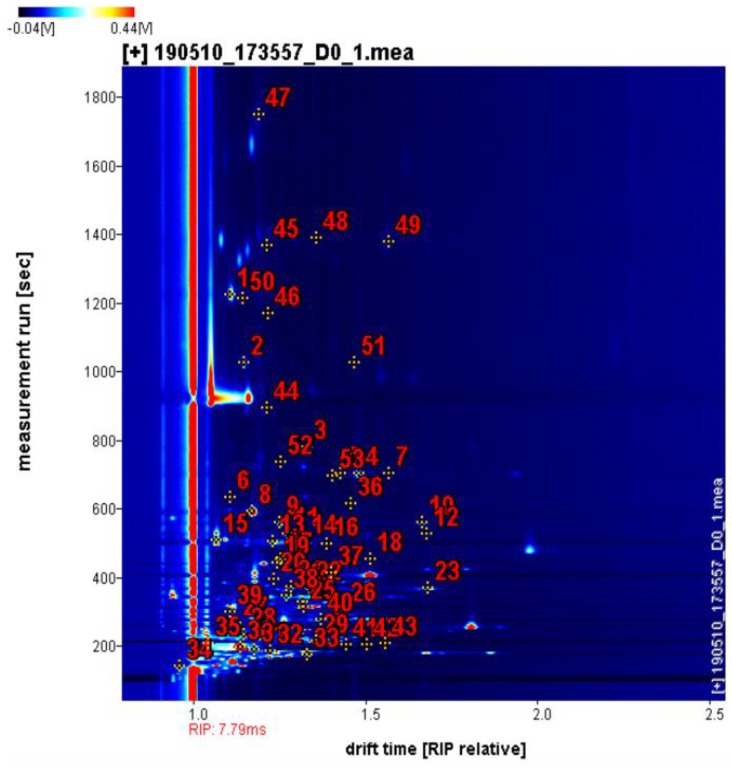
Ion migration spectra of jujube fruits stored for different times at 4 °C. The numbers are identified volatile components.

The aromatic components of fruits undergo many complicated changes during storage, such as vitamin degradation and phenol oxidation, resulting in changes in fruit flavor. Rodrigo et al. [34] found that the aroma of peaches was related to storage conditions and fruit quality characteristics. As the refrigeration progressed, the aroma of peaches dropped considerably, and the flavor and fruit sweetness, juiciness, and texture were strongly correlated. In this study, the volatile components of jujube fruits at different storage periods were determined by HS-GC-IMS. The samples were ionized in the column and then identified using ion mobility systems based on retention and drift times. The qualitative analysis of volatile components in jujube fruits is shown by numbers in Figure 1, where the ordinate represented the retention time, and the abscissa represented the drift time. A total of fifty-three peaks, forty-seven components were identified from the GC×IMS library (Figure 1 and Table 1), including fourteen alcohols, six aldehydes, nine esters, six ketones, two organic acids, two furans, three pyrazines, four sulfur-containing compounds, linalool oxide, and 2-methoxy-4-cresol. Among them, sulfur-containing compounds, linalool oxide, and 2-methoxy-4-cresol were detected in jujube fruits for the first time. This finding may be ascribed to the differences in detection methods and essential differences of raw materials. The identified components are listed in Table 1, which includes the compound name, CAS number, molecular formula, molecular weight, retention index, retention time, and drift time. Moreover, other substances with signals were detected but could not be determined were not listed. When moving through the drift region, due to the formation of adducts between the analyzed ions and neutral molecules (such as dimers and trimers), multiple signals were observed for a single compound [35]. The compounds of 1-octen-3-one, 3-hydroxy-2-butanone, 1-pentanol, heptanal, amyl acetate, and ethyl propanoate exhibited two peaks due to the presence of both monomer and dimer.

### 2.2. Differential Analysis of the Topographic Plots of Volatile Components in Jujube Fruits at Different Storage Periods

**Figure 2:**
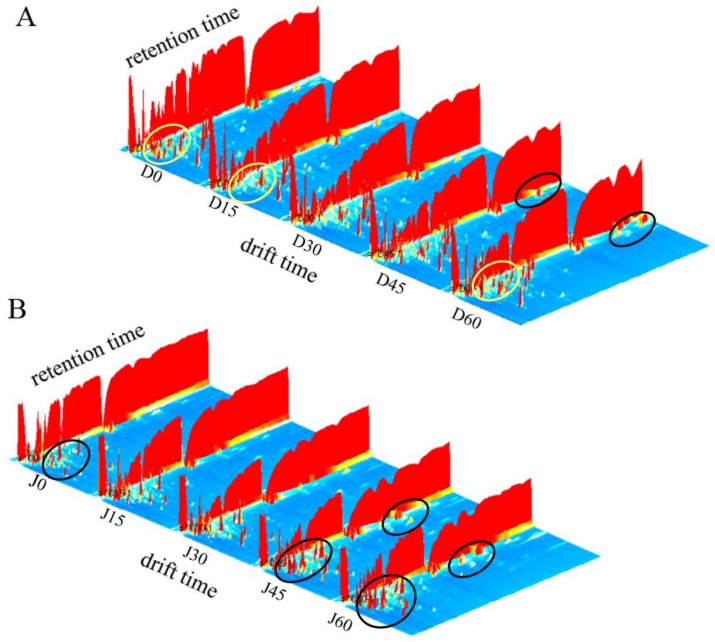
3D-topographic plots of jujube fruits at different times. Jujube fruits are Dongzao shown in (**A**), D0, D15, D30, D45, and D60 represent refrigeration for 0, 15, 30, 45, and 60 days, respectively. Jujube fruits are Jinsixiaozao shown in (**B**), J0, J15, J30, J45, and J60 represent refrigeration for 0, 15, 30, 45, and 60 days, respectively.

For an intuitive observation and comparison, topographic plots were used to characterize the substances of different jujube fruits during storage. From the 3D topographic plot (Figure 2), it can be clearly observed that with prolonged storage periods, the content of some compounds decreased, and new substances were formed. For DZ, some of the volatile components in the yellow circles disappeared after 15 days of storage but new peaks appeared after 60 days. Moreover, new peaks with high retention time were found after 45 days of storage, as indicated by the black circles (Figure 2A). For JS, more new peaks appeared when the storage time was extended to 45 days (Figure 2B). Hence, compared with fresh jujube fruits, volatile components were formed during the storage process [36]. During fruit ripening, the production of aroma volatiles (especially esters) was regulated by ethylene signaling pathways. Moreover, the production of volatile aroma components was strongly hampered in ethylene-suppressed fruits [37]. It could be inferred that after a certain time of refrigeration, the increase in ethylene synthesis led to more aroma components.

**Figure 3:**
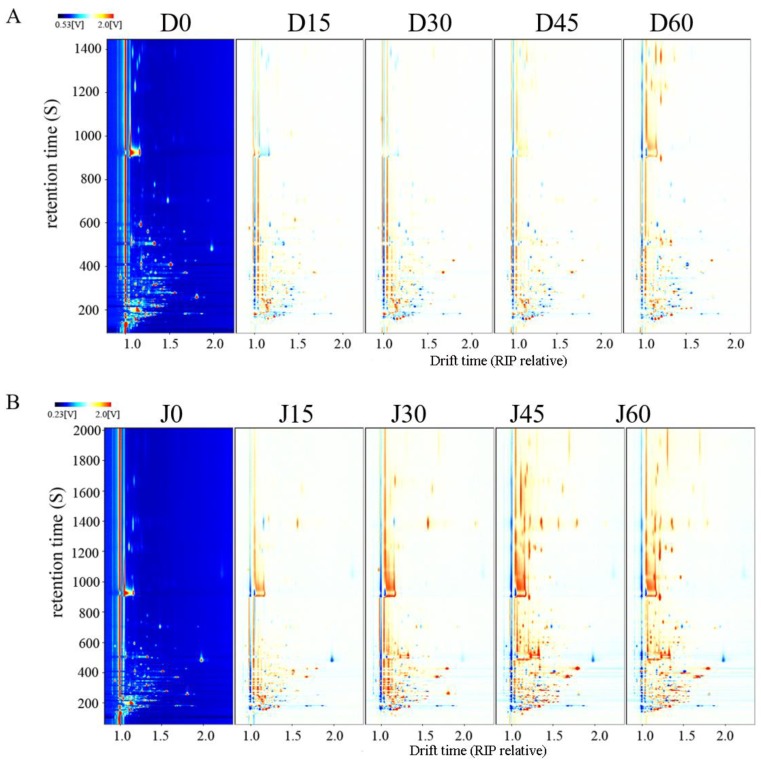
2D-topographic plots of jujube fruits at different times. Jujube fruits are Dongzao shown in (**A**), D0, D15, D30, D45, and D60 represent refrigeration for 0, 15, 30, 45, and 60 days, respectively. Jujube fruits are Jinsixiaozao shown in (**B**), J0, J15, J30, J45, and J60 represent refrigeration for 0, 15, 30, 45, and 60 days, respectively.

Considering that the 3D spectrum was rough, the overhead view was used for comparison, as represented in Figure 3. Different volatile organic components (VOCs) were different points in the picture, which was highly convenient for observation and analysis. The red vertical line at the abscissa was the reactive ion peak (RIP) at normalized drift times for DZ and JS of 7.79 and 7.81, respectively. The differential contrast model was used to compare the differences among the samples. The fresh sample was selected as the reference, and the spectrum of the other samples deducted the reference. The background after deduction was white showed that the VOCs were the same. Red spots indicate that the content of the substance was higher than the reference, and blue spots indicate that the content was lower than that of the reference. Compared with that of untreated fruits, more red spots are located in the retention time range of 900–1400 s of DZ after storage, and the VOCs changed inconspicuously in the retention time range of 100–600 s at the topographic plot (Figure 3A). For JS, many red spots appeared, and the entire retention time was covered. Especially after 45 days, most of the signals were much higher than that of fresh fruits. This finding indicated that the VOCs of two species varied at different refrigeration times and were affected by variety when the fruits were under similar conditions. Further ripening of the jujube fruits during storage promoted the synthesis of certain volatile components. The formation of fruit flavor was a dynamic process in which the fruit continuously synthesized volatile aroma substances [38]. In the post-harvest ripening stage of bananas, the ester content in the yellow ripening period increased, and the alcohol content in the over-ripe period increased [39].

### 2.3. Fingerprints of VOCs in Jujube Fruits at Different Storage Periods

**Figure 4:**
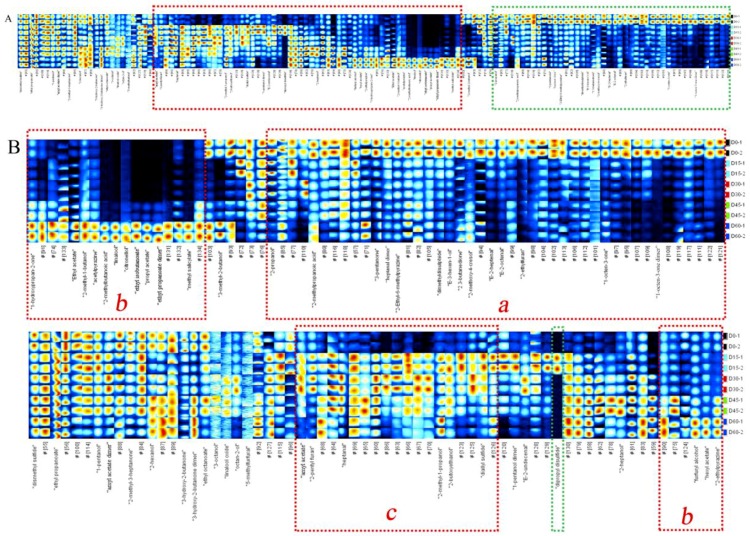
Fingerprint of volatile compounds of Dongzao samples. (**A**) Fingerprint of all Dongzao, (**B**) two parts of the fingerprint.

**Figure 5:**
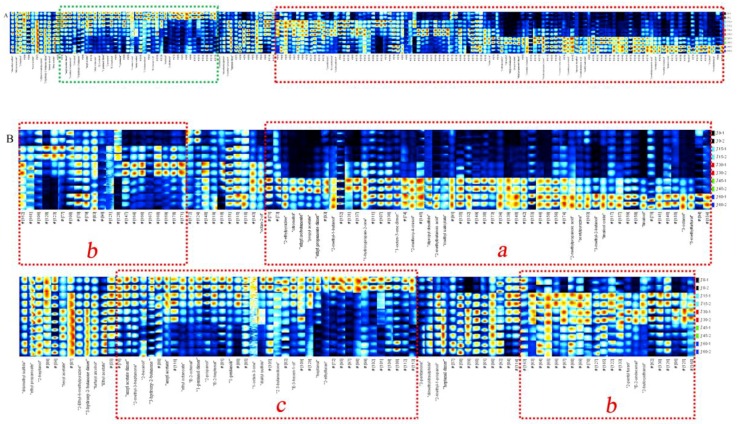
Fingerprint of volatile compounds of Jinsixiaozao samples. (**A**) Fingerprint of all Jinsixiaozao, (**B**) two parts of the fingerprint.

Although the topographic plots showed the tendency of volatile components, it is difficult to make an accurate judgment for the dense material on the map. The use of fingerprint was a good way to solve this problem. According to the peak signal of the topographic plots, the fingerprints of jujube fruits were formed (Figure 4 and Figure 5). In the fingerprints, each row represents the entire signal peak of one sample, and each column represents the same substance in different samples. Each cell represents the content of a substance at different times. Colors represent the content of volatile compounds. The brighter the color, the higher the content. Two compounds with the same name in the fingerprints were the monomers and their dimers. The drift time of dimers was increased due to their proton affinity and higher content [40]. By utilizing the fingerprints, the VOCs between different samples can be compared intuitively, moreover, the dynamic changes of each substance can be revealed. The unidentified substances are represented by numbers in the fingerprints. During the whole storage period, the volatile components detected in JS were more than those in DZ, which may be caused by the differences in the varieties and genetic factors of the two jujube fruits.

The VOCs of the jujube fruits constantly changed during storage. By comparing the intensity of spot for the profiles of VOCs at different stages, changes in the substances during storage (increased, decreased, disappeared or fluctuated) can be determined. Substances in the green-framed areas of Figure 4A and Figure 5A were the most abundant in fresh jujube fruits but dramatically decreased or even disappeared during the later period of storage. For DZ, 2-propanol, 3-methyl-2-butanol, 3-pentanone, and heptanal dimer were detected, while amyl acetate, ethyl octanoate, (*E*)-2-octenal, and 1-pentanol dimers were detected in JS. Simultaneously, it can be clearly seen that, compared to other periods, the relative amount percentage of these substances was the highest in fresh fruits during the entire storage period (Table S1). This finding may be due to the degradation of these substances during refrigeration.

By contrast, with prolonged storage time, some new compounds appeared, which showed strong signal intensity and bright color (the red-framed areas of Figure 4A and Figure 5A). These substances included linalool, ethyl isobutanoate, propyl acetate, ethyl ester, and methyl salicylate. This finding may be attributed to the complex physiological metabolism in fruits during storage, which mainly were a series of reactions, such as fatty acid, amino acid, and carbohydrate metabolism. Esters, which are mainly derived from the lipoxygenase pathway and amino acid metabolism are associated with the “fruity” attributes of fruit flavor, and its levels typically increase in the later periods of the ripening process [41]. Moreover, peaches stored after pre-storage were sweeter and had higher levels of propyl acetate, amyl acetate, and 2-methyl-1-butanol than control fruits [42]. The VOCs of the unframed part in the fingerprints presented few changes during the whole storage period, indicating that these substances were relatively stable during the cold storage of jujube fruits.

### 2.4. Changes of VOCs during Different Storage Periods

Changes in volatile components during different storage periods have been observed from the fingerprints. To compare the changes of each substance clearly, the fingerprint of each jujube fruit was divided into two parts based on the characteristic volatiles presented in different parts (Figure 4B and Figure 5B). The representative VOCs detected in fresh DZ and JS were found in the region *a* of Figure 4B and region *c* of Figure 5B, respectively. The same ingredients included 1-octen-3-one, 2-ethylfuran, (*E*)-2-octenal, (*E*)-2-heptenal, 2,3-butanedione, (*E*)-3-hexen-1-ol, and heptanal. Chen et al. [43] studied ten different varieties of Chinese jujube fruits and found that aldehydes had the highest content and contributed to the aroma of fresh jujube fruits, moreover, (*E*)-2-hexenal, hexanal, (*Z*)-2-heptenal, benzaldehyde, and (*E*)-2-nonenal were the common volatile components in fresh jujube fruits. This finding differed from our results, presumably due to differences in varieties and detection methods. Heredity can determine precursors, enzymes, and their activity in the formation of flavor components in jujube fruits. Compared with fresh jujube fruits, these parameters decreased significantly after 15 days of storage in DZ and 30 days in JS. Aldehydes significantly affected the flavor of fresh jujube fruits, which generated from the oxidation of fatty acids and the metabolism of amino acids [44]. They can affect the overall aroma of samples at low content for their low odor threshold values [45]. (*E*)-2-Heptenal was characterized by soap and almond flavor, (*E*)-2-octenal was associated with roasted, cucumber, nutty, and fatty characteristics, and heptanal has a fishy, nutty and sweet apricot note flavor [46].

Many different changes of VOCs have been observed during storage. As shown in Figure 4B, the contents of 3-methyl-2-butanol, heptanal dimer, 2-ethyl-6-methylpyrazine, dimethyldisulphide, (*E*)-3-hexen-1-ol, (*E*)-2-octenal, (*E*)-2-heptenal, 2-ethylfuran, and 1-octen-3-one dimer in fresh DZ were much higher than that after storage. During storage, their contents continuously declined, (*E*)-2-octenal, (*E*)-2-heptenal, 2-ethylfuran, and 1-octen-3-one dimer vanished after storage. It showed that 2-methylpropanoic acid, 3-pentanone, 2,3-butanedione, 2-methoxy-4-cresol, and 1-octen-3-one were important for the flavor of fresh DZ. The contents of 3-pentanone and 2,3-butanedione were initially the highest in DZ, which decreased significantly after a certain period of storage and increased slightly after 60 days, while in JS, 3-pentanone with low content in fresh fruits. Low-carbon saturated ketones have a special aroma, and many ketones have been found in cheese, which are the main volatile constituents of cheese with a unique flavor [47]. In addition, it can be observed that the change of 2-methylpropanoic acid was consistent with 3-pentanone and 2,3-butanedione. However, 2-methylbutanoic acid was different from them, it appeared with a strong signal at 60 days, which was not detected during the storage time of 0–45 days. Additionally, its relative amount percentage was up to 100% when stored for 60 days, before this period, this value was basically less than 10% (Table S1). Interestingly, only these two short-chain organic acids were identified in the two cultivars of jujube fruits. In JS, they appeared in storage for 45 days and then enhanced (Figure 5B). Organic acid is derived from the oxidation of fatty acids, which affects the flavor of jujube fruits [48]. During ripening, fruits undergo an esterification reaction, consuming a large amount of acid. When the jujube fruits are ripe, the amount of acids becomes low [49]. However, long-term storage resulted in the oxidation and decomposition of fatty acids. Besides, it should be noted that high content of organic acids can damage fruit quality and deteriorate the flavor of jujube fruits. In addition, processing methods also affect the content of organic acids. Chen et al. reported that acids were the major group among all the volatile chemicals in dried jujube fruits. Moreover, pre-treatment of jujube fruits with 5% CO_2_ can induce a decrease in acid content during drying, which is mainly caused by a decrease in lauric acid and nutmeg acid content [18]. 2-Methoxy-4-cresol was a newly detected component in the VOCs of jujube fruits, which appeared with high content in fresh DZ but not found in fresh JS and showed a pleasant clove-like flavor [50]. For DZ, its signal intensity was almost invisible after storage for 15–30 days, then gradually strengthened. In JS, it appeared after 45 days with a bright color in the fingerprint plot.

Some volatile compounds, mostly including esters, were rare in fresh samples of two jujube fruits but abundant in the later storage period, which were the most important volatile components at the end of storage (Figure 4B and Figure 5B), including hexyl acetate, ethyl acetate, ethyl propanoate, propyl acetate, ethyl isobutanoate, and methyl salicylate. Most of these compounds show a typical fruity or floral flavor, which may greatly affect the aroma of fruits. Zhou et al. found that low-temperature treatment could prevent the loss of aromatic esters during the ripening of ‘Nanguo’ pears at room temperature [51]. During the cold storage period, although complete ripening of the jujube fruits was inhibited, ripening proceeded at a slower pace, thus generating more esters. It can be found that the relative amount percentage of ethyl propanoate, propyl acetate, ethyl isobutanoate, and methyl salicylate was lower in the two cultivars of jujube at 0–30 days of storage and increased after 45 days. Moreover, it is worth noting that long-term storage at low temperatures inhibits the activity of enzymes, such as lipoxygenase and alcohol acyltransferase, which are the key enzymes in the aroma metabolism of fruits esters, resulting in a reduction in fruit flavor [52]. The jujube fruits in this investigation were refrigerated for 45–60 days, showed a good flavor. However, some substances had an adverse effect on the flavor of jujube fruits when the content was too high. The odor of high content of ethyl acetate was not described as “fruity” but rather as an off-flavor. Its elevated levels are usually associated with the over-ripeness and/or anaerobic metabolism of the fruit [53].

Two alcohols, including linalool and citronellol, showed the same variation tendency of the whole storage period, which was not detected at the initial stages of storage but appeared at a later time. For DZ they were detected with high signal intensity when the storage time was 60 days, but they appeared earlier in JS, showing a high content after 45 days of storage. This condition might have occurred because, with the prolongation of storage time, the glycoside precursors and other precursors for linalool and citronellol synthesis were formed in the jujube fruits. He et al. found that the content of linalool was closely related to the temperature of lemon-flavored hard tea during storage. At room temperature, its content increased slightly but decreased significantly when the temperature increased [54]. An interesting phenomenon was that the monoterpene alcohol of transgenic citrus peels could induce resistance against fungal invasion [55]. Therefore, whether jujube fruits were infected by fungi in the late storage period was uncertain, and the increased in linalool and citronellol content may be related to the self-defensive mechanism of fruits.

Meanwhile, 2-ethylpyrazine and acetylpyrazine with weak signal in fresh jujube fruits, and eye-catching signals have emerged in the fingerprints until storage for 45 days. Pyrazines were the characteristic flavor of the Maillard reaction and were often found in dried jujube fruits, which show a roasted or nutty flavor [56]. During drying, heat treatment could accelerate the progress of the Maillard reaction, resulting in more furans and pyrazines were formed. In this study, jujube fruits were stored at low temperature, which did not provide a good condition for the formation of pyrazines compared with drying.

Some substances with the highest content during the middle of storage. Amyl acetate, 2-pentyl furan, 2-butoxyethanol, diallyl sulfide, heptanal, 1-pentanol dimer, and (*E*)-2-undecenal were the characteristic VOCs when DZ was stored at 4 °C for 15–30 days. Among these compounds, 2-pentyl furan, 2-butoxyethanol, and (*E*)-2-undecenal were the characteristic VOCs in JS in the same period. Heptanal, 2-methyl-1-propanol, and 2-butoxyethanol remained in DZ and were maintained at a high content during the subsequent storage period. In addition, the content of furfuryl alcohol continued to increase during storage, reaching a maximum of 60 days. Linalool and citronellol were synthesized in large quantities at the late storage stage. Most alcohols showed increased contents in the later stages, indicating that aldehydes were reduced and converted into corresponding alcohols at this stage, thus promoting the esterification of alcohols with acids produced by anaerobic respiration during storage. However, the signal intensity of 2-butoxyethanol disappeared later in JS, but the heptanal dimer was largely accumulated.

Dipropyl disulfide was only detected when the storage period was 15 days in DZ samples, but its content increased in JS after storage for 45 days. Three other sulfide compounds, namely, dimethyl sulfide, diallyl sulfide, and dimethyldisulphide were identified. The signal of dimethyl sulfide was observed in the whole storage process, and minor changes in signal intensity were observed in all samples. Diallyl sulfide and dimethyldisulphide were the characteristic VOCs of fresh JS and DZ, respectively. Sulfur-containing compounds are widely found in vegetables [57]. Diallyl sulfide was the characteristic flavor of garlic, dipropyl disulfide was associated with onion, and dimethyldisulphide was abundant in the volatile components of cabbage. Sulfur-containing compounds commonly arise from sulfur-bearing precursors, and in jujube fruits, sulfur-containing amino acids primarily exist, such as methionine. Low content of sulfur compounds can enhance the aromatic flavor of jujube fruits. Pyrazines and sulfur-containing compounds had low odor thresholds, and they acted with other compounds to enhance the overall aroma in jujube fruits [58,59]. Some substances, such as ethyl propanoate, amyl acetate dimer, 2-methyl-3-heptanone, and 3-hydroxy-2-butanone, existed in all periods of two jujube fruits, and the signal had minor changes.

### 2.5. PCA of Jujube Fruits at Different Storage Periods

**Figure 6:**
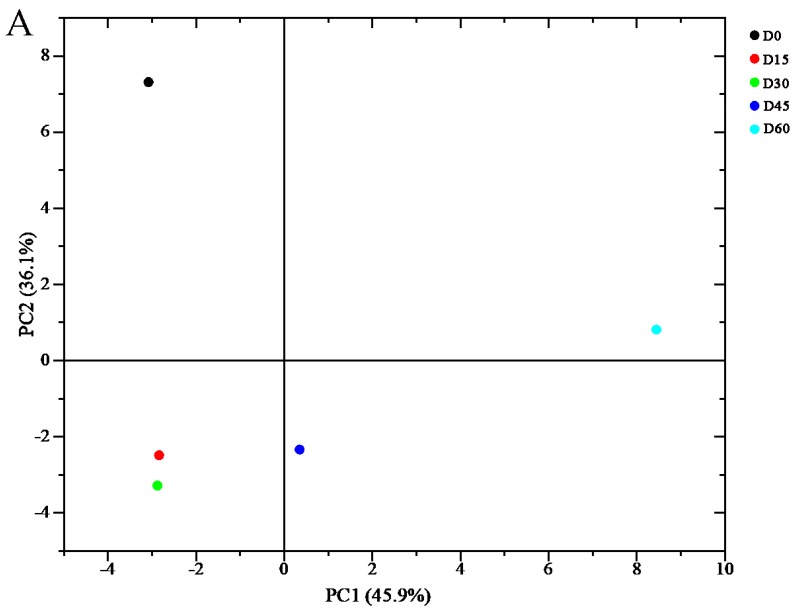

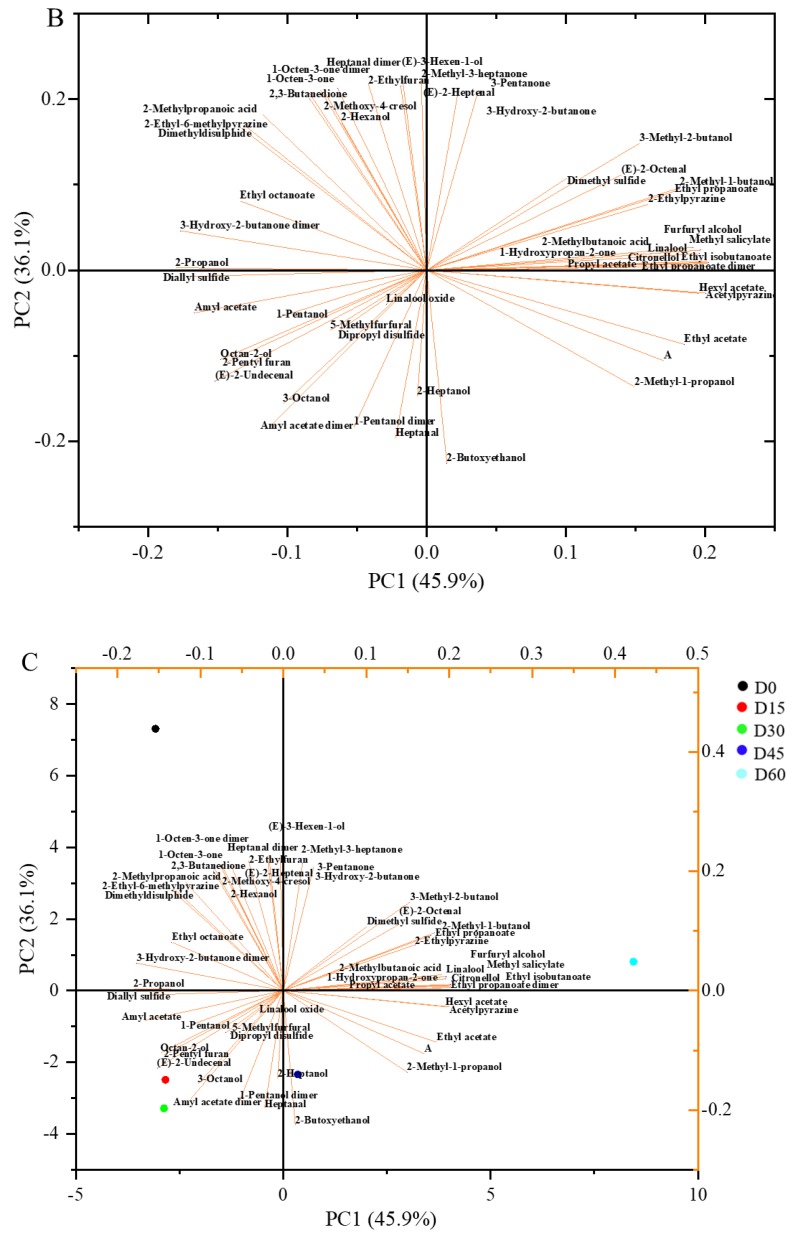
PCA analysis of Dongzao. (**A**) score plot of the first two principal components, D0, D15, D30, D45, and D60 represent refrigeration for 0, 15, 30, 45, and 60 days, respectively. (**B**) loading plot of different variances, (**C**) biplot of PCA.

**Figure 7:**
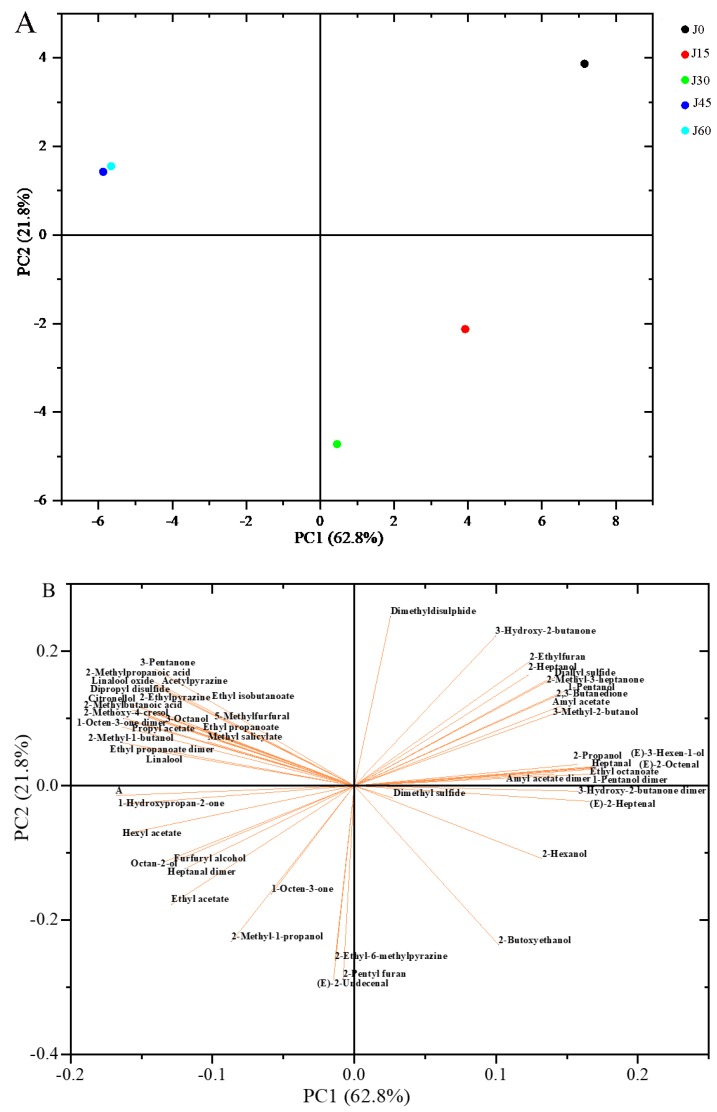

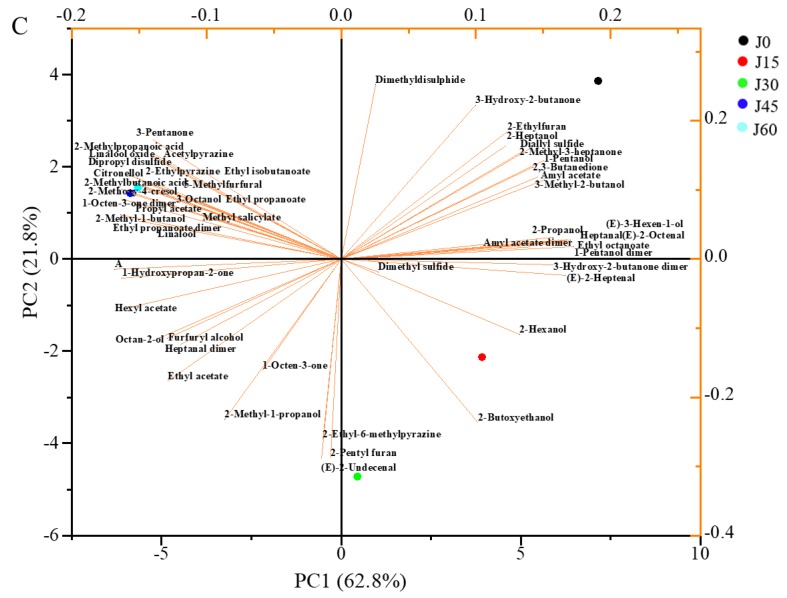
PCA analysis of Jinsixiaozao. (**A**) Score plot of the first two principal components, J0, J15, J30, J45, and J60 represent refrigeration for 0, 15, 30, 45, and 60 days, respectively. (**B**) Loading plot of different variances, (**C**) biplot of PCA.

PCA is a multivariate statistical analysis method that uses multiple variables to linearly transform to select a few significant variables. The main features are extracted for linear analysis by reducing the dimensionality of the data, and the main information is retained in several unrelated principal components [60]. Generally, when the cumulative contribution rate reaches 60%, PCA model is selected as the separation model [61]. Li et al. found the volatile components of different tissue parts of tomato showed disparate distributions among four varieties by PCA [62]. In this study, PCA was performed to analyzed the variation of 53 identified volatile compounds in the two cultivars of jujube. Firstly, all data were normalized to calculate the covariance matrix and its eigenvalues and eigenvectors, which derived from the corresponding peak area of each volatile component of jujube fruits. Then, the principal component was determined, and the corresponding contribution rate was calculated. Finally, a classification program was adopted in a smaller space to illustrate the relationship of jujube fruits during different storage periods. The results were shown in Figure 6 and Figure 7, a clear separation trend of jujube fruits at different storage periods in two principal components can be observed.

As shown in Figure 6 and Figure 7, the first principal component (PC1) and the second principal component (PC2) explained 82% and 84.6% of the total variables of the model in DZ and JS, respectively. A remarkable difference was found between fresh jujube fruits and refrigerated ones in volatile components. On the axis, a large distance is found between fresh DZ and the other samples, the four other samples in different storage periods can be distinguished easily. Among them, the ones stored for 15 days and 30 days are closer (Figure 6A), which indicates that the volatile components of DZ in these two periods are similar. In JS, changes of the sample are distributed from right to left in the PCA, as the storage time was extended (Figure 7A). Jujube fruits stored for 0–30 days are distributed in the right quadrant, while the ones stored for 45–60 days are in the left quadrant with a higher similarity. Based on the PCA, the jujube fruits at different periods of two cultivars are separated well. Additionally, some information on the volatile compounds was lost during the statistical re-modeling. As shown in the loading plots (Figure 6B and Figure 7B), the length of the arrow reflects the extent of information loss, the shorter the arrow, the more information lost [20]. For example, linalool oxide and 5-methyfurfural suffered the most with the loss of their information of DZ, but in JS, dimethyl sulfide loss was the highest.

To get more details, the biplots were used (Figure 6C and Figure 7C). From the biplots, it can be clearly seen that 2-methoxy-4-cresol, heptanal and some ketones (1-octen-3-one and 2,3-butanedione) were positively related to the fresh DZ. However, for JS, 3-hydroxy-2-butanone contributed a lot to the flavor of the fresh fruits. When storage for 15 days, (*E*)-undecenal and 2-penty furanl were closely related to the DZ, 2-butoxyethanol was positively related to JS. In the biplots, the relationship between specific volatile components and jujube fruits in a certain period was demonstrated. While at the end of the storage, most esters were positively related to the jujube fruits, while 2-methylbutanoic acid and 2-methylpropanoic acid were found to be positively related to JS. These results were in accordance with the above fingerprints.

### 2.6. Cluster Analysis of VOCs of Jujube Fruits from Different Periods Based on the Heat Map

**Figure 8:**
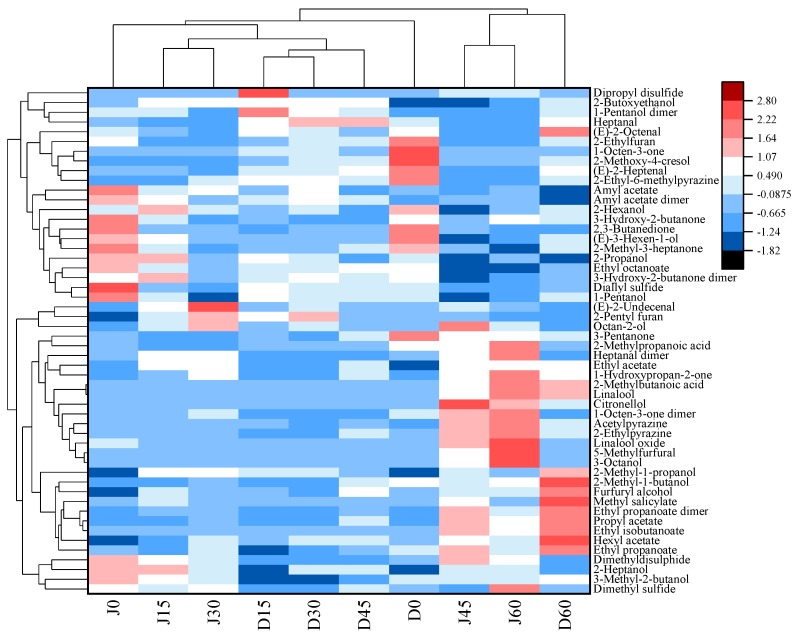
Heat map and cluster analysis of jujube fruits at different periods. D0, D15, D30, D45, and D60 represent DZ refrigerated for 0, 15, 30, 45, and 60 days, respectively; J0, J15, J30, J45, and J60 represent JS refrigerated for 0, 15, 30, 45, and 60 days, respectively.

To further understand the differences in VOCs of two jujube fruits at different storage periods, cluster analysis was performed using a heat map (Figure 8). According to the vertical direction of the heat map, all samples were classified into two main categories. Jujube fruits from JS that were stored for 45 and 60 days and those from DZ that were stored for 60 days were clustered together to form a group (J45, J60, and D60 in Figure 8), the rest of the samples were clustered together to form another group (J0 to J30, and D0 to D45 in Figure 8). At the late period of storage, the volatile components of DZ were very similar to those in JS and were quite different from those in the early storage period. The second group can also be divided into four categories. Among them, fresh jujube fruits from different cultivars were clustered into two different groups. Jujube fruits from JS, which were stored for 15 and 30 days, were grouped together (J15 and J30 in Figure 8), and those from DZ were stored for 15, 30 and 45 days, which were from the same class (D15, D30, and D45 in Figure 8).

According to the above results, we can infer that the volatile components of DZ and JS largely differ due to the differences in their varieties. The content of 1-octen-3-one and 2-methoxy-4-cresol was higher in fresh DZ than that in JS. Samples from D60, J45, and J60 were similar in 2-pentyl furan, 1-octen-3-one, ethyl acetate and propyl acetate content. Cluster analysis show that the storage time greatly influenced the volatile components of jujube fruits, and the flavor of jujube fruits varied during different periods. This finding was consistent with the fingerprint and PCA.

## 3. Materials and Methods

### 3.1. Experimental Materials

Two cultivars of jujube fruits were used for this experiment, namely, *Zizyphus jujuba* Mill. *cv*. ‘Dongzao’ and *Zizyphus jujuba* Mill. *cv*. ‘Jinsixiaozao’. Fresh DZ were harvested in September 2018, from a garden in Zhanhua County, Shandong province, China (118°7′56″ E, 37°4′53″ N). JS were harvested from an experimental field station in Qidong County, Hengyang City, Hunan Province, China (111°59′14″ E, 26°48′3″ N). After harvest, they were transported to the laboratory within 24 h with a cold chain. Jujube fruits with intact appearance, similar morphological properties, and free from visible blemish, disease, and mechanical injury were selected as raw materials. Samples were stored at 4 °C with a humidity of 90% and obtained every 15 days until storage for 60 days. The collected samples (500 g) were de-nucleated and freeze-dried, then the dried jujube fruits were crushed into powder with a grinder (RoyalstarRS-FS1401, Zhongshan Rongshida Kitchen & Bathroom Appliance Co., Ltd., Zhongshan, China), after that, collected samples were passed through a 60-mesh sieve, sealed, and stored at −80 °C for subsequent analyses.

### 3.2. Apparatuses

Vacuum freeze dryer LGJ-25C (Beijing Sihuan Instrument Factory Co., Ltd. Beijing, China), HS-GC-IMS instrument: the GC-IMS FlavourSpec^®^ Gesellschaft für Analytische Sensorsysteme mbH (G.A.S., Dortmund, Germany). The device was equipped with an autosampler unit (CTC Analytics AG, Zwingen, Switzerland).

### 3.3. HS-GC-IMS Analysis

Analyses of jujube fruits were performed using the HS-GC-IMS instrument as described by Xin et al. with slight modifications [31]. Specifically, 1.0 g of fine powder was weighed and placed into a 20 mL headspace-glass sampling vial. The samples were incubated at 60 °C for 10 min. After incubation, 500 μL of headspace was automatically injected into the injector under splitless injection mode with a syringe at 85 °C. The GC was performed with an RTX-WAX (30 m, 0.53 mm ID, 1 μm film thickness, Restek Co., Bellefonte, PA, USA) capillary column to separate volatile components and coupled to IMS at 45 °C. Nitrogen (99.999% purity) was used as the carrier gas under the following programmed flow: 2 mL/min for 2 min and maintenance for 8 min, 20 mL/min for 10 min and 50 mL/min for 15 min, and flow then stopped. The instrument was performed under ambient pressure. The analytes were separated at 40 °C in the column and then ionized in the IMS ionization chamber of 45 °C. The drift gas (nitrogen gas) was set at 150 mL/min.

In this experiment, the instrument was standardized with n-ketones whose retention index was linear, because IMS had no response to alkanes. The retention index (RI) of volatile compounds was calculated by using n-ketones C4-C9 (Sinopharm Chemical Reagent Beijing Co., Ltd., Beijing, China) as external references. Volatile compounds were identified by comparing RI and the drift time (the time it takes for ions to reach the collector through drift tube, in milliseconds) of standard in the GC-IMS library (Gesellschaft für Analytische Sensorsysteme mbH, Dortmund, Germany).

### 3.4. Data Analysis

The instrumental analysis software included laboratory analytical viewer (LAV, G.A.S., Dortmund, Germany), three plug-ins (G.A.S., Dortmund, Germany), and GC × IMS library search, which can be used for sample analysis from different perspectives. The spectra were analyzed using the LAV software, and the difference profiles and fingerprints of volatile components were constructed using the Reporter and Gallery plug-ins. The NIST and IMS databases were built into the software for qualitative analysis of the materials. The principal component analysis (PCA) and heat map were used for clustering analysis of samples. The heat map and PCA were generated using Origin 2018 software (OriginLab, Northampton, MA, USA).

## 4. Conclusions

In this study, 47 volatile compounds were identified in jujube fruits at different storage periods at 4 °C via HS-GC-IMS. Among the identified substances, alcohols, esters, ketones and aldehydes were predominant, and a few organic acids were found. At the same time, some new compounds were found, namely, four sulfur-containing compounds (dimethyldisulphide, dimethyl sulfide, dipropyl disulfide, diallyl sulfide), linalool oxide, and 2-methoxy-4-cresol. The differences of the volatile compounds at different cold storage times between varieties and individuals were revealed by using topographic plots and fingerprints. Aldehydes and ketones were the main volatile components in fresh fruits and the early storage, while esters changed obviously and became the main VOCs at the end of the storage, which synthesized in large quantities at 45 days (JS) and 60 days (DZ), respectively. According to the PCA and heat map, the samples from different periods were well separated, the stored jujube fruits kept a long distance from the fresh one on the PCA map. The results showed that HS-GC-IMS could identify the characteristic volatile compounds of jujube fruits. It could provide an efficient method for determining the storage period of jujube fruits due to simple sample preparation steps and shorter analysis time.

## Figures and Tables

**Table 1:** The information on identified compounds of jujube fruits.

NO.	Compound	CAS#	Formula	MW	RI	Rt (Sec)	Dt (RIP Relative)	Comment	Identification Approach
1	Furfuryl alcohol	C98000	C_5_H_6_O_2_	98.1	1661.4	1226.886	1.1116		RI, Dt
2	Octan-2-ol	C123966	C_8_H_18_O	130.2	1401.0	704.688	1.4313		RI, Dt
3	(*E*)-3-Hexen-1-ol	C928972	C_6_H_12_O	100.2	1327.8	562.241	1.2529		RI, Dt
4	2-Butoxyethanol	C111762	C_6_H_14_O_2_	118.2	1400.9	704.400	1.5715		RI, Dt
5	2-Heptanol	C543497	C_7_H_16_O	116.2	1291.4	500.190	1.3920		RI, Dt
6	1-Pentanol	C71410	C_5_H_12_O	88.1	1262.0	456.411	1.2576	monomer	RI, Dt
7	1-Pentanol	C71410	C_5_H_12_O	114.2	1262.0	456.411	1.5164	dimer	RI, Dt
8	2-Methyl-1-butanol	C137326	C_5_H_12_O	88.1	1215.7	397.445	1.2367		RI, Dt
9	2-Hexanol	C626937	C_6_H_14_O	102.2	1194.9	374.160	1.2880		RI, Dt
10	3-Methyl-2-butanol	C598754	C_5_H_12_O	88.1	1124.2	306.504	1.4417		RI, Dt
11	2-Propanol	C67630	C_3_H_8_O	60.1	908.5	188.339	1.2264		RI, Dt
12	2-Methyl-1-propanol	C78831	C_4_H_10_O	74.1	1090.2	279.704	1.3727		RI, Dt
13	Linalool	C78706	C_10_H_18_O	154.3	1496.8	896.913	1.2161		RI, Dt
14	Citronellol	C106229	C_10_H_20_O	156.3	1742.8	1390.307	1.3610		RI, Dt
15	3-Octanol	C589980	C_8_H_18_O	130.2	1397.5	697.657	1.4073		RI, Dt
16	(*E*)-2-Octenal	C2548870	C_8_H_14_O	126.2	1438.5	779.930	1.3345		RI, Dt
17	(*E*)-2-Heptenal	C18829555	C_7_H_12_O	112.2	1326.9	560.684	1.6667		RI, Dt
18	Heptanal	C111717	C_7_H_14_O	114.2	1191.5	370.381	1.3412	monomer	RI, Dt
19	Heptanal	C111717	C_7_H_14_O	114.2	1193.2	372.270	1.6841	dimer	RI, Dt
20	3-Methylbutanal	C590863	C_5_H_10_O	86.1	917.4	190.741	1.1796		RI, Dt
21	(*E*)-2-undecenal	C53448070	C_11_H_20_O	168.3	1736.9	1378.477	1.5700		RI, Dt
22	5-Methylfurfural	C620020	C_6_H_6_O_2_	110.1	1562.0	1027.52	1.4726		RI, Dt
23	1-Octen-3-one	C4312996	C_8_H_14_O	126.2	1308.2	527.716	1.2773	monomer	RI, Dt
24	1-Octen-3-one	C4312996	C_8_H_14_O	126.2	1308.2	527.716	1.6826	dimer	RI, Dt
25	1-Hydroxypropan-2-one	C116096	C_3_H_6_O_2_	74.1	1295.3	506.307	1.2352		RI, Dt
26	3-Hydroxy-2-butanone	C513860	C_4_H_8_O_2_	88.1	1298.6	511.779	1.0710	monomer	RI, Dt
27	3-Hydroxy-2-butanone	C513860	C_4_H_8_O_2_	88.1	1295.5	506.629	1.3273	dimer	RI, Dt
28	2,3-Butanedione	C431038	C_4_H_6_O_2_	86.1	1021.6	233.375	1.1507		RI, Dt
29	3-Pentanone	C96220	C_5_H_10_O	86.1	989.2	215.961	1.3600		RI, Dt
30	2-Methyl-3-heptanone	C13019200	C_8_H_16_O	128.2	1169.0	347.292	1.2730		RI, Dt
31	Ethyl octanoate	C106321	C_10_H_20_O_2_	172.3	1401.0	704.688	1.4841		RI, Dt
32	Amyl acetate	C628637	C_7_H_14_O_2_	130.2	1150.4	329.460	1.3196	monomer	RI, Dt
33	Amyl acetate	C628637	C_7_H_14_O_2_	130.2	1134.6	315.334	1.3240	dimer	RI, Dt
34	Ethyl propanoate	C105373	C_5_H_10_O_2_	102.1	942.6	197.947	1.1418	monomer	RI, Dt
35	Ethyl acetate	C141786	C_4_H_8_O_2_	88.1	866.3	176.930	1.3333		RI, Dt
36	Hexyl acetate	C142927	C_8_H_16_O_2_	144.2	1230.8	415.502	1.4044		RI, Dt
37	Ethyl propanoate	C105373	C_5_H_10_O_2_	102.1	964.6	205.502	1.4486	dimer	RI, Dt
38	Propyl acetate	C109604	C_5_H_10_O_2_	102.1	968.8	207.090	1.5065		RI, Dt
39	Ethyl isobutanoate	C97621	C_6_H_12_O_2_	116.2	970.1	207.619	1.5604		RI, Dt
40	Methyl salicylate	C119368	C_8_H_8_O_3_	152.1	1732.1	1368.733	1.2162		RI, Dt
41	2-Methylpropanoic acid	C79312	C_4_H_8_O_2_	88.1	1562.1	1027.802	1.1494		RI, Dt
42	2-Methylbutanoic acid	C116530	C_5_H_10_O_2_	102.1	1632.9	1169.846	1.2188		RI, Dt
43	2-Ethylpyrazine	C13925003	C_6_H_8_N_2_	108.1	1365.8	634.530	1.1116		RI, Dt
44	2-Ethyl-6-methylpyrazine	C13925036	C_7_H_10_N_2_	122.2	1345.4	594.936	1.1737		RI, Dt
45	Acetylpyrazine	C22047252	C_6_H_6_N_2_O	122.1	1654.4	1212.857	1.1463		RI, Dt
46	2-Pentyl furan	C3777693	C_9_H_14_O	130.2	1253.1	444.179	1.2477		RI, Dt
47	2-Ethylfuran	C3208160	C_6_H_8_O	96.1	975.6	209.900	1.0454		RI, Dt
48	Dimethyldisulphide	C624920	C_2_H_6_S_2_	94.2	1074.6	268.202	1.1262		RI, Dt
49	Dimethyl sulfide	C75183	C_2_H_6_S	62.1	737.5	142.102	0.9636		RI, Dt
50	Dipropyl disulfide	C629196	C_6_H_14_S_2_	150.3	1356.5	616.330	1.4603		RI, Dt
51	Diallyl sulfide	C592881	C_6_H_10_S	114.2	1118.4	301.687	1.1088		RI, Dt
52	Linalool oxide	C60047178	C_10_H_18_O_2_	170.3	1417.2	737.207	1.2585		RI, Dt
53	2-Methoxy-4-cresol	C93516	C_8_H_10_O_2_	138.2	1921.7	1749.150	1.1921		RI, Dt

MW: molecular mass; RI: retention index; Rt: retention time; Dt: drift time.

## Supplementary Materials

The following are available online, Table S1: Relative amount percentage (%) of the same compound at different refrigerated times.

## Abbreviations

HS-GC-IMS	headspace-gas chromatography-ion mobility spectrometry
DZ	*Zizyphus jujuba* Mill. *cv.* Dongzao
JS	*Zizyphus jujuba* Mill. *cv*. Jinsixiaozao
VOCs	volatile organic compounds
PCA	PCA principal component analysis
