# Supplementary material for: Characterization of Volatile Component Changes in Jujube Fruits during Cold Storage by Using Headspace-Gas Chromatography-Ion Mobility Spectrometry
Source: Molecules. 2019 Oct 30;24(21):3904. doi: 10.3390/molecules24213904 (PMC6864690; doi:10.3390/molecules24213904)
Supplement: Supplementary file 1 [file molecules-24-03904-s001.pdf]

Table S1. Relative amount percentage (%) of the same compound at different refrigerated times.

Compound	Relative amount percentage (%)																			
	Dongzao										Jinsixiaozao									
	D0-1	D0-2	D15-1	D15-2	D30-1	D30-2	D45-1	D45-2	D60-1	D60-2	C0-1	C0-2	C15-1	C15-2	C30-1	C30-2	C45-1	C45-2	C60-1	C60-2
Furfuryl alcohol	73	73	72	66	60	70	83	86	100	96	79	66	96	95	90	87	100	98	91	96
Octan-2-ol	62	62	66	66	100	96	66	77	44	37	17	14	41	44	74	76	95	100	53	52
(E)-3-hexen-1-ol	90	100	66	68	49	52	37	39	40	39	98	100	85	82	45	50	17	17	26	25
2-Butoxyethanol	31	40	93	96	90	100	87	87	71	84	67	48	96	90	100	96	32	35	41	35
2-Heptanol	42	44	48	52	84	86	99	100	59	52	100	94	87	94	64	63	77	74	79	67
1-Pentanol	85	90	100	94	82	85	92	94	84	79	94	100	68	67	40	39	39	39	42	45
1-Pentanol dimer	26	28	100	97	66	70	59	58	45	50	92	100	91	85	47	47	29	28	26	26
2-Methyl-1-butanol	54	56	35	18	22	22	51	53	95	100	30	35	26	27	47	51	70	76	66	100
2-Hexanol	91	100	71	70	81	80	59	57	73	75	76	84	98	100	80	74	54	53	64	72
3-Methyl-2-butanol	65	68	2	2	5	6	33	33	95	100	98	100	86	83	58	62	54	53	70	63
2-Propanol	82	87	99	100	86	88	43	43	30	24	100	87	96	91	43	48	23	20	43	46
2-Methyl-1-propanol	25	25	71	61	70	69	58	60	100	95	35	33	100	93	95	98	90	87	78	78
Linalool	4	5	2	2	3	3	9	8	97	100	27	25	14	14	14	16	62	62	79	100
Citronellol	15	15	15	13	13	13	25	47	88	100	8	4	4	4	5	4	81	100	62	56
3-Octanol	57	71	85	85	71	92	92	100	50	64	9	13	13	8	14	14	52	50	100	90
(E)-2-Octenal	75	100	50	49	39	41	26	27	22	19	94	100	84	85	45	63	29	29	31	36
(E)-2-Heptenal	78	100	56	62	28	34	13	16	18	18	84	90	100	86	45	63	29	22	27	31

Table S1. *Cont.*

Compound	Relative amount percentage (%)																			
	Dongzao										Jinsixiaozao									
	D0-1	D0-2	D15-1	D15-2	D30-1	D30-2	D45-1	D45-2	D60-1	D60-2	C0-1	C0-2	C15-1	C15-2	C30-1	C30-2	C45-1	C45-2	C60-1	C60-2
Heptanal	40	43	69	68	96	99	98	100	60	63	92	100	57	54	55	55	19	17	16	15
Heptanal dimer	100	100	56	48	56	58	47	46	56	55	22	26	62	62	66	69	54	54	84	100
(E)-2-Undecenal	40	46	72	72	100	97	55	53	14	23	9	8	63	63	100	97	42	45	29	29
5-Methylfurfural	61	76	69	84	69	61	100	92	61	53	9	7	7	7	7	7	46	45	98	100
1-Octen-3-one	87	100	32	34	20	21	36	38	67	67	45	72	100	45	54	81	63	63	72	72
1-Octen-3-one dimer	60	100	10	19	12	8	10	10	10	12	20	21	17	17	35	35	85	87	100	99
1-Hydroxypropan-2-one	43	45	36	33	42	44	70	73	95	100	39	35	45	44	70	71	75	77	88	100
3-Hydroxy-2-butanone	95	100	79	80	66	67	69	71	90	85	90	100	68	67	58	56	61	60	73	74
3-Hydroxy-2-butanone dimer	96	100	80	76	95	98	84	83	56	63	93	85	99	100	50	51	23	22	32	50
2,3-Butanedione	98	100	55	54	38	36	42	43	70	66	87	100	40	40	40	38	31	34	20	23
3-Pentanone	98	100	51	51	39	38	66	67	69	70	58	59	41	41	44	43	98	100	97	97
2-Methyl-3-heptanone	98	100	76	76	69	70	79	76	80	82	100	99	75	78	59	57	68	70	53	55
Ethyl octanoate	95	100	79	80	71	66	97	91	49	58	92	100	75	68	54	45	13	11	14	22
Amyl acetate	62	66	60	69	92	100	43	53	21	34	98	100	57	59	80	81	43	39	50	52

Table S1. *Cont.*

Compound	Relative amount percentage (%)																			
	Dongzao										Jinsixiaozao									
	D0-1	D0-2	D15-1	D15-2	D30-1	D30-2	D45-1	D45-2	D60-1	D60-2	C0-1	C0-2	C15-1	C15-2	C30-1	C30-2	C45-1	C45-2	C60-1	C60-2
Amyl acetate dimer	62	65	88	85	98	100	86	88	60	62	97	100	90	90	66	67	73	73	63	68
Ethyl propanoate	82	74	58	58	68	68	75	74	99	100	73	74	66	66	71	78	94	96	99	100
Ethyl acetate	18	18	40	41	47	46	71	71	100	99	29	30	93	93	97	97	98	99	98	100
Hexyl acetate	45	43	47	50	54	57	58	52	83	100	53	47	60	63	84	85	100	98	98	69
Ethyl propanoate dimer	1	1	1	1	2	2	22	22	94	100	2	2	14	13	20	20	95	100	48	47
Propyl acetate	1	1	1	1	2	2	26	26	95	100	1	1	3	3	10	9	95	100	57	69
Ethyl isobutanoate	1	1	1	1	1	1	20	20	100	100	2	1	2	2	3	3	91	100	51	65
Methyl salicylate	7	5	7	7	6	7	9	10	98	100	32	22	55	52	22	24	100	98	44	45
2-Methylpropanoic acid	100	93	62	59	61	59	46	47	45	47	38	41	33	25	30	28	72	71	100	95
2-Methylbutanoic acid	5	5	7	5	5	5	9	13	97	100	11	6	10	11	19	22	76	85	97	100
2-Ethylpyrazine	63	61	20	25	22	20	84	86	100	98	10	13	9	10	13	15	76	74	91	100
2-Ethyl-6-methylpyrazine	94	100	68	70	72	75	61	64	58	60	63	64	75	77	94	100	66	65	75	78
Acetylpyrazine	23	25	36	38	26	40	45	52	100	94	35	26	18	19	18	15	70	74	92	100
2-Pentyl furan	52	53	87	86	94	100	44	44	34	33	19	21	73	69	95	100	52	52	34	34
2-Ethylfuran	95	100	27	28	33	31	41	41	24	25	100	100	30	28	26	26	22	21	29	34
Dimethyldisulphide	97	100	68	65	48	49	42	45	73	47	100	81	73	68	35	38	86	71	70	72

Table S1. *Cont.*

Compound	Relative amount percentage (%)																			
	Dongzao										Jinsixiaozao									
	D0-1	D0-2	D15-1	D15-2	D30-1	D30-2	D45-1	D45-2	D60-1	D60-2	C0-1	C0-2	C15-1	C15-2	C30-1	C30-2	C45-1	C45-2	C60-1	C60-2
Dimethyl sulfide	94	97	77	85	86	79	100	100	100	92	84	75	69	76	77	86	52	63	80	100
Dipropyl disulfide	5	7	100	98	7	4	5	5	6	7	25	19	13	27	16	25	100	94	91	94
Diallyl sulfide	84	90	100	89	73	74	88	90	47	48	98	100	36	31	17	20	14	17	10	18
Linalool oxide	66	73	53	53	60	93	100	93	60	60	27	25	14	14	14	16	62	62	79	100
2-Methoxy-4-cresol	100	93	48	31	37	41	62	75	75	82	15	15	11	11	26	23	91	96	98	100

In the table, D0 (C0), D15 (C15), D30 (C30), D45 (C45), and D60 (C60) represented refrigeration for 0, 15, 30, 45, and 60 days, respectively. When the compound has the maximum content on the overall storage period, its relative amount percentage is considered to be 100%, and then the relative amount percentage of the substance in each period is calculated based on the maximum content. Finally, the changes of various substances in different periods can be determined.
